# (2,2′-Bipyridine-κ^2^ *N*,*N*′){*N*-[(2-oxidonaphthalen-1-yl-κ*O*)methyl­idene]-l-valinato-κ*O*}copper(II) trihydrate

**DOI:** 10.1107/S1600536812022702

**Published:** 2012-05-26

**Authors:** Jian Zuo, Caifeng Bi, Yuhua Fan, Nan Zhang, Pengfei Zhang

**Affiliations:** a Key Laboratory of Marine Chemistry Theory and Technology, Ministry of Education, College of Chemistry and Chemical Engineering, Ocean University of China, Qingdao, Shandong 266100, People’s Republic of China

## Abstract

In the title complex, [Cu(C_16_H_15_NO_3_)(C_10_H_8_N_2_)]·3H_2_O, the Cu^II^ atom is five coordinated by *O*,*N*,*O*′-donor atoms of the Schiff base ligand and by two N atoms of the 2,2′-bipyridine ligand in a distorted square-pyramidal geometry. In the crystal, mol­ecules are linked into a two-dimensional network parallel to (011) by O—H⋯O hydrogen bonds.

## Related literature

For general background to Schiff base ligands in coordination chemistry, see: Garnovski *et al.* (1993 ▶); Yamada (1999 ▶). For the properties of Schiff base complexes, see: Kano *et al.* (2003 ▶); Mukherjee *et al.* (2009 ▶).
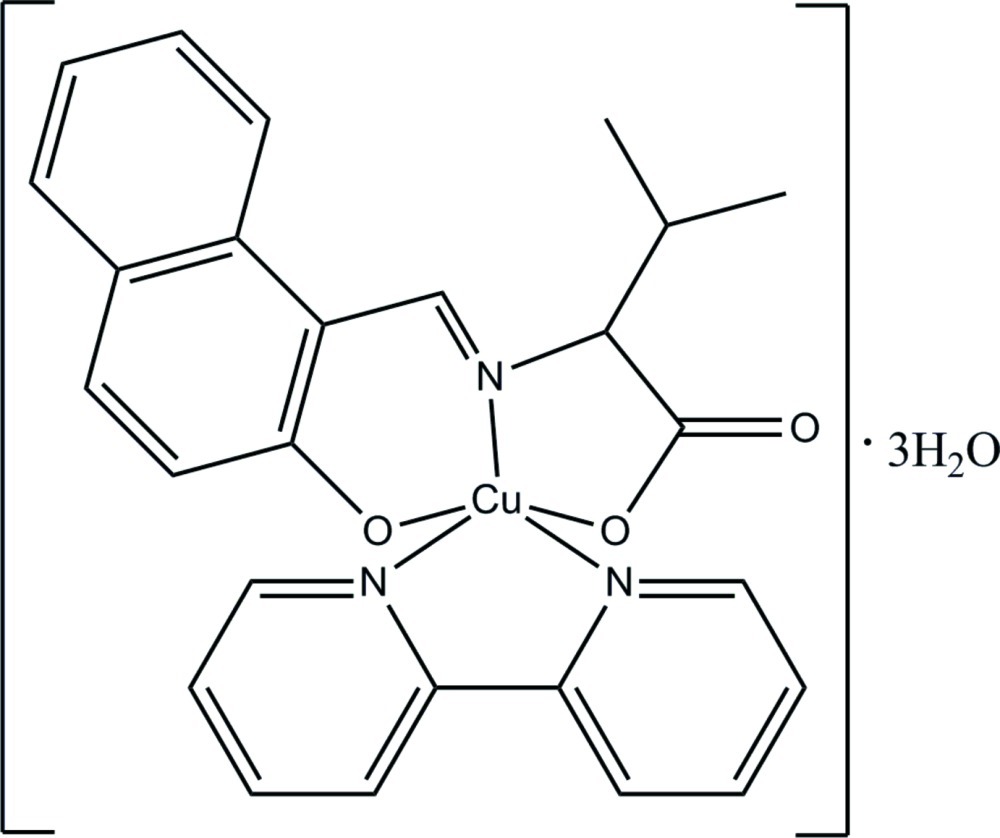


## Experimental

### 

#### Crystal data

[Cu(C_16_H_15_NO_3_)(C_10_H_8_N_2_)]·3H_2_O
*M* *_r_* = 543.06
Triclinic, 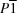

*a* = 9.295 (1) Å
*b* = 9.7861 (11) Å
*c* = 14.3819 (15) Å
α = 79.971 (1)°
β = 74.718 (1)°
γ = 85.745 (2)°
*V* = 1242.1 (2) Å^3^
*Z* = 2
Mo *K*α radiation
μ = 0.93 mm^−1^
*T* = 298 K
0.46 × 0.43 × 0.42 mm

#### Data collection

Bruker SMART CCD area-detector diffractometer
Absorption correction: multi-scan (*SADABS*; Bruker, 2002 ▶) *T*_min_ = 0.675, *T*_max_ = 0.697
6423 measured reflections
4306 independent reflections
3246 reflections with *I* > 2σ(*I*)
*R*_int_ = 0.022

#### Refinement

*R*[*F* ^2^ > 2σ(*F* ^2^)] = 0.039
*wR*(*F* ^2^) = 0.097
*S* = 1.04
4306 reflections
327 parameters
H-atom parameters constrained
Δρ_max_ = 0.39 e Å^−3^
Δρ_min_ = −0.27 e Å^−3^

### 

Data collection: *SMART* (Bruker, 2002 ▶); cell refinement: *SAINT* (Bruker, 2002 ▶); data reduction: *SAINT*; program(s) used to solve structure: *SHELXS97* (Sheldrick, 2008 ▶); program(s) used to refine structure: *SHELXL97* (Sheldrick, 2008 ▶); molecular graphics: *SHELXTL* (Sheldrick, 2008 ▶); software used to prepare material for publication: *SHELXTL*.

## Supplementary Material

Crystal structure: contains datablock(s) I, global. DOI: 10.1107/S1600536812022702/bx2410sup1.cif

Structure factors: contains datablock(s) I. DOI: 10.1107/S1600536812022702/bx2410Isup2.hkl

Additional supplementary materials:  crystallographic information; 3D view; checkCIF report

## Figures and Tables

**Table 1:** Hydrogen-bond geometry (Å, °)

*D*—H⋯*A*	*D*—H	H⋯*A*	*D*⋯*A*	*D*—H⋯*A*
O4—H4*A*⋯O2^i^	0.85	1.99	2.823 (3)	168
O4—H4*B*⋯O3	0.85	2.00	2.847 (3)	172
O5—H5*A*⋯O2^ii^	0.85	2.07	2.870 (3)	158
O5—H5*B*⋯O6^iii^	0.85	2.04	2.852 (4)	160
O6—H6*A*⋯O4^iv^	0.85	2.10	2.900 (4)	156
O6—H6*B*⋯O5^v^	0.85	1.97	2.814 (4)	174

Symmetry codes: (i) 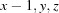; (ii) 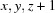; (iii) 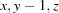; (iv) 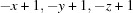; (v) 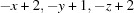.

**Fig. 1.**
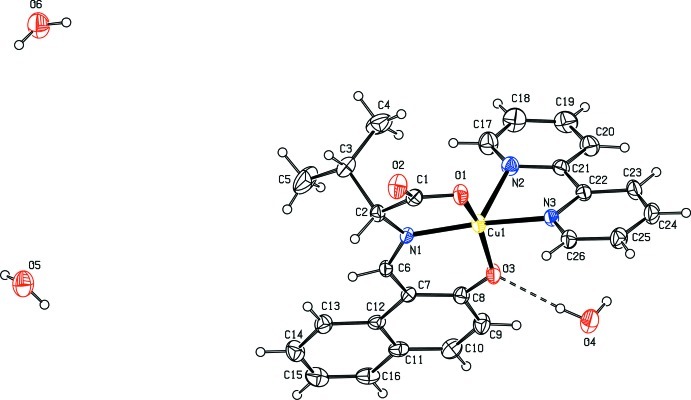
The structure of the title complex, showing 30% probability displacement ellipsoids and the atom-numbering scheme.

**Fig. 2.**
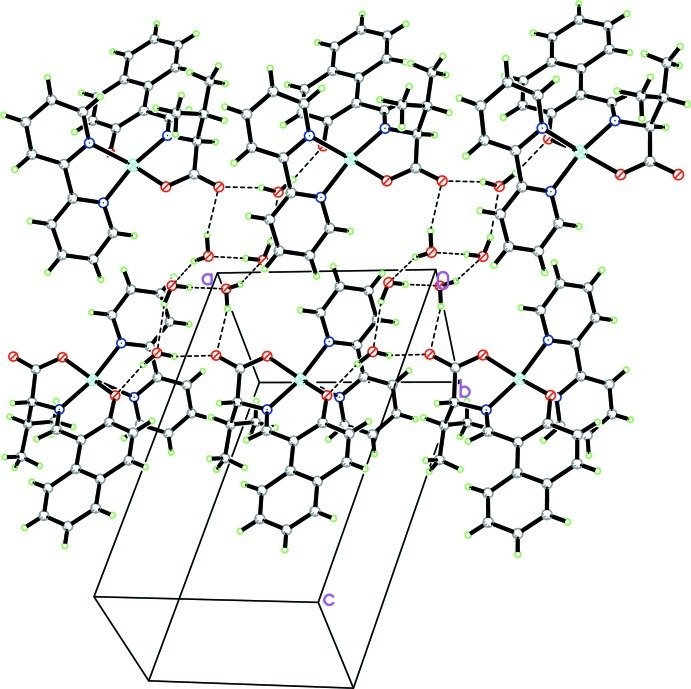
Two dimensional network of the title complex.

## supplementary crystallographic information

### Comment

Schiff base ligands represent one of the most widely utilized classes of ligands in coordination chemistry (Garnovski *et al.*, 1993), and their metal complexes have been studied for many years (Yamada, 1999). These complexes have been used in the fields of magnetism, catalysis and enzymatic reactions (Kano *et al.*, 2003; Mukherjee *et al.*, 2009). In this paper, we report the synthesis and crystal structure of a copper complex with Schiff base and 2,2'-bipyridine ligand.

The molecular structure of the title complex is shown in Fig. 1. The Cu atom is in a distorted square-pyramidal coordination geometry, defined by one N and two O atoms from the Schiff base ligand and two N atoms from a 2,2'-bipyridine ligand. The basal plane is formed by the atoms O1, O3, N1 and N3, their mean deviation from this plane is 0.0891 Å, and the Cu atom just out of this plane by 0.252 Å. The axial position of the pyramid is occupied by atom N2. In the crystal, molecules are linked into two-dimensional network by intermolecular O—H···O hydrogen bonds (Fig. 2).

### Experimental

2-Hydroxy-1-Naphthaldehyde (0.172 g, 1 mmol) was added to a methanol solution (60 ml) containing *L*-Valine (0.117 g, 1 mmol) and potassium hydroxide (0.056 g, 1 mmol). The mixture was stirred at 333 K for 3 h, then an aqueous solution (6 ml) of cupric acetate monohydrate (0.199 g, 1 mmol) was added dropwise and stirred for 2 h. A methanol solution (6 ml) of 2,2'-Bipyridine (0.156 g, 1 mmol) was then added dropwise and the mixture stirred for 2 h. The resulting green solution was allowed to evaporate slowly at room temperature for two weeks, yielding green block crystals.

### Refinement

All H-atoms were positioned geometrically and refined using a riding model, with the following constraints: C—H = 0.93 Å, *U*_iso_(H) =1.2*U*_eq_(C) for C*_sp_*2, C—H = 0.98 Å, *U*_iso_(H) =1.2*U*_eq_(C) for CH, C—H = 0.96 Å, *U*_iso_(H) =1.5*U*_eq_(C) for CH_3_, O—H = 0.85 Å, *U*_iso_(H) =1.2*U*_eq_(O) for OH.

### Crystal data

**Table:**

[Cu(C_16_H_15_NO_3_)(C_10_H_8_N_2_)]·3H_2_O	*Z* = 2
*M**_r_* = 543.06	*F*(000) = 566
Triclinic, *P*1	*D*_x_ = 1.452 Mg m^−^^3^
Hall symbol: -P 1	Mo *K*α radiation, λ = 0.71073 Å
*a* = 9.295 (1) Å	Cell parameters from 2185 reflections
*b* = 9.7861 (11) Å	θ = 2.8–23.9°
*c* = 14.3819 (15) Å	µ = 0.93 mm^−^^1^
α = 79.971 (1)°	*T* = 298 K
β = 74.718 (1)°	Block, green
γ = 85.745 (2)°	0.46 × 0.43 × 0.42 mm
*V* = 1242.1 (2) Å^3^	

### Data collection

**Table:**

Bruker SMART CCD area-detector diffractometer	4306 independent reflections
Radiation source: fine-focus sealed tube	3246 reflections with *I* > 2σ(*I*)
Graphite monochromator	*R*_int_ = 0.022
phi and ω scans	θ_max_ = 25.0°, θ_min_ = 3.0°
Absorption correction: multi-scan (*SADABS*; Bruker, 2002)	*h* = −11→10
*T*_min_ = 0.675, *T*_max_ = 0.697	*k* = −11→11
6423 measured reflections	*l* = −12→17

### Refinement

**Table:**

Refinement on *F*^2^	Primary atom site location: structure-invariant direct methods
Least-squares matrix: full	Secondary atom site location: difference Fourier map
*R*[*F*^2^ > 2σ(*F*^2^)] = 0.039	Hydrogen site location: inferred from neighbouring sites
*wR*(*F*^2^) = 0.097	H-atom parameters constrained
*S* = 1.04	*w* = 1/[σ^2^(*F*_o_^2^) + (0.0382*P*)^2^ + 0.5836*P*] where *P* = (*F*_o_^2^ + 2*F*_c_^2^)/3
4306 reflections	(Δ/σ)_max_ = 0.001
327 parameters	Δρ_max_ = 0.39 e Å^−^^3^
0 restraints	Δρ_min_ = −0.27 e Å^−^^3^

### Special details

**Table:**

Geometry. All e.s.d.'s (except the e.s.d. in the dihedral angle between two l.s. planes) are estimated using the full covariance matrix. The cell e.s.d.'s are taken into account individually in the estimation of e.s.d.'s in distances, angles and torsion angles; correlations between e.s.d.'s in cell parameters are only used when they are defined by crystal symmetry. An approximate (isotropic) treatment of cell e.s.d.'s is used for estimating e.s.d.'s involving l.s. planes.
Refinement. Refinement of *F*^2^ against ALL reflections. The weighted *R*-factor *wR* and goodness of fit *S* are based on *F*^2^, conventional *R*-factors *R* are based on *F*, with *F* set to zero for negative *F*^2^. The threshold expression of *F*^2^ > σ(*F*^2^) is used only for calculating *R*-factors(gt) *etc*. and is not relevant to the choice of reflections for refinement. *R*-factors based on *F*^2^ are statistically about twice as large as those based on *F*, and *R*- factors based on ALL data will be even larger.

### Fractional atomic coordinates and isotropic or equivalent isotropic displacement parameters (Å^2^)

**Table:**

	*x*	*y*	*z*	*U*_iso_*/*U*_eq_	
Cu1	0.54013 (4)	0.29935 (4)	0.23335 (3)	0.03351 (14)	
N1	0.6348 (2)	0.2715 (2)	0.34020 (17)	0.0276 (5)	
N2	0.3955 (3)	0.4995 (3)	0.22238 (19)	0.0396 (6)	
N3	0.4708 (3)	0.3005 (3)	0.11297 (17)	0.0324 (6)	
O1	0.7456 (2)	0.3182 (2)	0.15227 (14)	0.0398 (5)	
O2	0.9811 (2)	0.2916 (3)	0.15860 (16)	0.0507 (6)	
O3	0.3595 (2)	0.2123 (2)	0.31584 (14)	0.0400 (5)	
O4	0.1797 (3)	0.0875 (3)	0.22315 (18)	0.0628 (7)	
H4A	0.1222	0.1558	0.2101	0.075*	
H4B	0.2397	0.1189	0.2493	0.075*	
O5	1.0190 (3)	0.2087 (3)	0.97155 (18)	0.0662 (7)	
H5A	0.9955	0.2526	1.0197	0.079*	
H5B	0.9419	0.1648	0.9746	0.079*	
O6	0.8146 (3)	1.0165 (3)	0.95467 (19)	0.0689 (8)	
H6A	0.8096	1.0114	0.8972	0.083*	
H6B	0.8707	0.9509	0.9737	0.083*	
C1	0.8461 (3)	0.2996 (3)	0.1986 (2)	0.0346 (7)	
C2	0.7965 (3)	0.2931 (3)	0.3090 (2)	0.0316 (7)	
H2	0.8473	0.2135	0.3396	0.038*	
C3	0.8416 (4)	0.4271 (3)	0.3358 (2)	0.0452 (8)	
H3	0.9502	0.4318	0.3107	0.054*	
C4	0.7762 (4)	0.5558 (4)	0.2846 (3)	0.0714 (13)	
H4C	0.6693	0.5528	0.3037	0.107*	
H4D	0.8109	0.5593	0.2152	0.107*	
H4E	0.8071	0.6369	0.3024	0.107*	
C5	0.8084 (5)	0.4242 (5)	0.4448 (3)	0.0878 (15)	
H5C	0.8571	0.4994	0.4577	0.132*	
H5D	0.8445	0.3376	0.4747	0.132*	
H5E	0.7027	0.4336	0.4712	0.132*	
C6	0.5780 (3)	0.2084 (3)	0.4268 (2)	0.0298 (7)	
H6	0.6435	0.1803	0.4660	0.036*	
C7	0.4246 (3)	0.1769 (3)	0.4694 (2)	0.0284 (7)	
C8	0.3236 (3)	0.1816 (3)	0.4110 (2)	0.0333 (7)	
C9	0.1733 (3)	0.1428 (4)	0.4575 (2)	0.0451 (8)	
H9	0.1062	0.1436	0.4196	0.054*	
C10	0.1265 (4)	0.1050 (3)	0.5553 (3)	0.0464 (9)	
H10	0.0271	0.0828	0.5831	0.056*	
C11	0.2239 (4)	0.0982 (3)	0.6167 (2)	0.0371 (7)	
C12	0.3742 (3)	0.1334 (3)	0.5741 (2)	0.0317 (7)	
C13	0.4680 (4)	0.1241 (3)	0.6382 (2)	0.0412 (8)	
H13	0.5675	0.1473	0.6131	0.049*	
C14	0.4150 (4)	0.0817 (4)	0.7363 (2)	0.0506 (9)	
H14	0.4800	0.0750	0.7763	0.061*	
C15	0.2672 (4)	0.0484 (4)	0.7777 (2)	0.0521 (9)	
H15	0.2329	0.0208	0.8447	0.062*	
C16	0.1735 (4)	0.0567 (3)	0.7188 (2)	0.0450 (8)	
H16	0.0740	0.0347	0.7462	0.054*	
C17	0.3553 (4)	0.5916 (4)	0.2821 (3)	0.0521 (9)	
H17	0.4092	0.5928	0.3280	0.062*	
C18	0.2377 (4)	0.6866 (4)	0.2804 (3)	0.0604 (10)	
H18	0.2146	0.7516	0.3226	0.072*	
C19	0.1567 (4)	0.6813 (4)	0.2144 (3)	0.0577 (10)	
H19	0.0762	0.7428	0.2116	0.069*	
C20	0.1939 (4)	0.5860 (4)	0.1527 (3)	0.0503 (9)	
H20	0.1388	0.5815	0.1080	0.060*	
C21	0.3148 (3)	0.4960 (3)	0.1574 (2)	0.0365 (7)	
C22	0.3657 (3)	0.3928 (3)	0.0911 (2)	0.0329 (7)	
C23	0.3139 (4)	0.3911 (4)	0.0096 (2)	0.0457 (9)	
H23	0.2417	0.4560	−0.0053	0.055*	
C24	0.3696 (4)	0.2929 (4)	−0.0490 (2)	0.0492 (9)	
H24	0.3330	0.2890	−0.1027	0.059*	
C25	0.4792 (4)	0.2009 (4)	−0.0281 (2)	0.0461 (9)	
H25	0.5201	0.1350	−0.0679	0.055*	
C26	0.5271 (3)	0.2084 (3)	0.0532 (2)	0.0386 (8)	
H26	0.6021	0.1465	0.0675	0.046*	

### Atomic displacement parameters (Å^2^)

**Table:**

	*U*^11^	*U*^22^	*U*^33^	*U*^12^	*U*^13^	*U*^23^
Cu1	0.0338 (2)	0.0408 (2)	0.0286 (2)	−0.00009 (16)	−0.01315 (16)	−0.00514 (16)
N1	0.0273 (13)	0.0314 (14)	0.0270 (13)	−0.0036 (10)	−0.0095 (11)	−0.0070 (11)
N2	0.0389 (15)	0.0396 (16)	0.0432 (16)	0.0040 (12)	−0.0146 (13)	−0.0104 (13)
N3	0.0348 (14)	0.0367 (15)	0.0273 (13)	−0.0032 (12)	−0.0116 (11)	−0.0028 (11)
O1	0.0360 (12)	0.0569 (14)	0.0283 (11)	−0.0049 (10)	−0.0110 (10)	−0.0056 (10)
O2	0.0308 (13)	0.0765 (18)	0.0449 (14)	−0.0010 (11)	−0.0045 (11)	−0.0184 (12)
O3	0.0334 (12)	0.0581 (15)	0.0311 (12)	−0.0085 (10)	−0.0141 (10)	−0.0015 (10)
O4	0.0614 (17)	0.0731 (18)	0.0638 (17)	−0.0118 (13)	−0.0305 (14)	−0.0114 (14)
O5	0.0723 (18)	0.079 (2)	0.0531 (16)	0.0036 (15)	−0.0199 (14)	−0.0236 (14)
O6	0.0751 (19)	0.0676 (18)	0.0730 (19)	0.0152 (14)	−0.0312 (16)	−0.0233 (15)
C1	0.0339 (18)	0.0337 (18)	0.0380 (18)	−0.0014 (14)	−0.0105 (15)	−0.0083 (14)
C2	0.0298 (16)	0.0342 (17)	0.0333 (17)	0.0019 (13)	−0.0124 (13)	−0.0066 (13)
C3	0.041 (2)	0.050 (2)	0.049 (2)	−0.0096 (16)	−0.0097 (16)	−0.0181 (17)
C4	0.067 (3)	0.039 (2)	0.117 (4)	0.0012 (19)	−0.031 (3)	−0.024 (2)
C5	0.112 (4)	0.103 (4)	0.061 (3)	−0.043 (3)	−0.017 (3)	−0.040 (3)
C6	0.0339 (17)	0.0314 (17)	0.0285 (17)	0.0009 (13)	−0.0148 (13)	−0.0069 (13)
C7	0.0320 (16)	0.0266 (16)	0.0288 (16)	−0.0015 (13)	−0.0089 (13)	−0.0084 (13)
C8	0.0318 (17)	0.0348 (18)	0.0341 (18)	0.0002 (13)	−0.0092 (14)	−0.0070 (14)
C9	0.0353 (19)	0.060 (2)	0.042 (2)	−0.0069 (16)	−0.0125 (16)	−0.0076 (17)
C10	0.0338 (19)	0.051 (2)	0.052 (2)	−0.0061 (16)	−0.0030 (16)	−0.0127 (17)
C11	0.046 (2)	0.0258 (17)	0.0362 (18)	−0.0024 (14)	−0.0023 (15)	−0.0086 (14)
C12	0.0421 (18)	0.0222 (15)	0.0311 (17)	−0.0022 (13)	−0.0063 (14)	−0.0087 (13)
C13	0.047 (2)	0.045 (2)	0.0328 (18)	−0.0051 (16)	−0.0095 (15)	−0.0100 (15)
C14	0.070 (3)	0.053 (2)	0.0316 (19)	0.0014 (19)	−0.0160 (18)	−0.0113 (16)
C15	0.075 (3)	0.045 (2)	0.0284 (18)	−0.0003 (19)	0.0009 (19)	−0.0079 (16)
C16	0.052 (2)	0.0311 (18)	0.043 (2)	−0.0018 (16)	0.0054 (17)	−0.0090 (15)
C17	0.053 (2)	0.057 (2)	0.052 (2)	0.0083 (19)	−0.0185 (18)	−0.0188 (19)
C18	0.059 (2)	0.058 (3)	0.067 (3)	0.014 (2)	−0.015 (2)	−0.026 (2)
C19	0.043 (2)	0.049 (2)	0.079 (3)	0.0135 (18)	−0.015 (2)	−0.010 (2)
C20	0.042 (2)	0.049 (2)	0.062 (2)	0.0049 (17)	−0.0207 (18)	−0.0040 (19)
C21	0.0326 (17)	0.0347 (18)	0.0406 (19)	−0.0053 (14)	−0.0126 (15)	0.0053 (14)
C22	0.0287 (16)	0.0386 (18)	0.0300 (16)	−0.0066 (14)	−0.0084 (13)	0.0023 (14)
C23	0.041 (2)	0.054 (2)	0.045 (2)	−0.0048 (17)	−0.0246 (17)	0.0061 (18)
C24	0.056 (2)	0.063 (2)	0.0331 (19)	−0.0151 (19)	−0.0196 (17)	−0.0029 (18)
C25	0.052 (2)	0.057 (2)	0.0329 (18)	−0.0079 (18)	−0.0125 (16)	−0.0113 (16)
C26	0.0428 (19)	0.0415 (19)	0.0350 (18)	−0.0014 (15)	−0.0154 (15)	−0.0076 (15)

### Geometric parameters (Å, º)

**Table:**

Cu1—N1	1.934 (2)	C7—C8	1.409 (4)
Cu1—O3	1.939 (2)	C7—C12	1.450 (4)
Cu1—O1	1.961 (2)	C8—C9	1.429 (4)
Cu1—N3	1.999 (2)	C9—C10	1.351 (5)
Cu1—N2	2.298 (3)	C9—H9	0.9300
N1—C6	1.283 (3)	C10—C11	1.413 (4)
N1—C2	1.471 (3)	C10—H10	0.9300
N2—C17	1.319 (4)	C11—C12	1.411 (4)
N2—C21	1.349 (4)	C11—C16	1.414 (4)
N3—C26	1.337 (4)	C12—C13	1.415 (4)
N3—C22	1.342 (4)	C13—C14	1.365 (4)
O1—C1	1.270 (3)	C13—H13	0.9300
O2—C1	1.238 (3)	C14—C15	1.384 (5)
O3—C8	1.306 (3)	C14—H14	0.9300
O4—H4A	0.8500	C15—C16	1.354 (5)
O4—H4B	0.8500	C15—H15	0.9300
O5—H5A	0.8499	C16—H16	0.9300
O5—H5B	0.8500	C17—C18	1.383 (5)
O6—H6A	0.8499	C17—H17	0.9300
O6—H6B	0.8499	C18—C19	1.368 (5)
C1—C2	1.523 (4)	C18—H18	0.9300
C2—C3	1.545 (4)	C19—C20	1.362 (5)
C2—H2	0.9800	C19—H19	0.9300
C3—C5	1.511 (5)	C20—C21	1.385 (4)
C3—C4	1.516 (5)	C20—H20	0.9300
C3—H3	0.9800	C21—C22	1.477 (4)
C4—H4C	0.9600	C22—C23	1.382 (4)
C4—H4D	0.9600	C23—C24	1.371 (5)
C4—H4E	0.9600	C23—H23	0.9300
C5—H5C	0.9600	C24—C25	1.368 (5)
C5—H5D	0.9600	C24—H24	0.9300
C5—H5E	0.9600	C25—C26	1.371 (4)
C6—C7	1.430 (4)	C25—H25	0.9300
C6—H6	0.9300	C26—H26	0.9300
			
N1—Cu1—O3	92.02 (9)	O3—C8—C7	124.4 (3)
N1—Cu1—O1	83.66 (9)	O3—C8—C9	117.1 (3)
O3—Cu1—O1	159.67 (9)	C7—C8—C9	118.4 (3)
N1—Cu1—N3	168.99 (10)	C10—C9—C8	121.5 (3)
O3—Cu1—N3	91.75 (9)	C10—C9—H9	119.2
O1—Cu1—N3	89.24 (9)	C8—C9—H9	119.2
N1—Cu1—N2	114.65 (10)	C9—C10—C11	122.0 (3)
O3—Cu1—N2	85.89 (9)	C9—C10—H10	119.0
O1—Cu1—N2	113.99 (9)	C11—C10—H10	119.0
N3—Cu1—N2	75.94 (10)	C12—C11—C10	118.6 (3)
C6—N1—C2	118.8 (2)	C12—C11—C16	120.1 (3)
C6—N1—Cu1	125.5 (2)	C10—C11—C16	121.3 (3)
C2—N1—Cu1	113.63 (17)	C11—C12—C13	116.7 (3)
C17—N2—C21	117.9 (3)	C11—C12—C7	119.7 (3)
C17—N2—Cu1	130.5 (2)	C13—C12—C7	123.6 (3)
C21—N2—Cu1	109.3 (2)	C14—C13—C12	121.2 (3)
C26—N3—C22	118.7 (3)	C14—C13—H13	119.4
C26—N3—Cu1	120.6 (2)	C12—C13—H13	119.4
C22—N3—Cu1	120.6 (2)	C13—C14—C15	121.8 (3)
C1—O1—Cu1	115.39 (19)	C13—C14—H14	119.1
C8—O3—Cu1	126.99 (18)	C15—C14—H14	119.1
H4A—O4—H4B	105.7	C16—C15—C14	118.9 (3)
H5A—O5—H5B	105.8	C16—C15—H15	120.6
H6A—O6—H6B	109.9	C14—C15—H15	120.6
O2—C1—O1	123.5 (3)	C15—C16—C11	121.4 (3)
O2—C1—C2	118.9 (3)	C15—C16—H16	119.3
O1—C1—C2	117.5 (3)	C11—C16—H16	119.3
N1—C2—C1	107.9 (2)	N2—C17—C18	123.9 (3)
N1—C2—C3	113.4 (2)	N2—C17—H17	118.1
C1—C2—C3	109.2 (2)	C18—C17—H17	118.1
N1—C2—H2	108.7	C19—C18—C17	117.5 (4)
C1—C2—H2	108.7	C19—C18—H18	121.2
C3—C2—H2	108.7	C17—C18—H18	121.2
C5—C3—C4	112.4 (3)	C20—C19—C18	120.0 (3)
C5—C3—C2	112.8 (3)	C20—C19—H19	120.0
C4—C3—C2	111.7 (3)	C18—C19—H19	120.0
C5—C3—H3	106.5	C19—C20—C21	119.1 (3)
C4—C3—H3	106.5	C19—C20—H20	120.4
C2—C3—H3	106.5	C21—C20—H20	120.4
C3—C4—H4C	109.5	N2—C21—C20	121.5 (3)
C3—C4—H4D	109.5	N2—C21—C22	115.9 (3)
H4C—C4—H4D	109.5	C20—C21—C22	122.6 (3)
C3—C4—H4E	109.5	N3—C22—C23	120.8 (3)
H4C—C4—H4E	109.5	N3—C22—C21	115.6 (3)
H4D—C4—H4E	109.5	C23—C22—C21	123.5 (3)
C3—C5—H5C	109.5	C24—C23—C22	119.5 (3)
C3—C5—H5D	109.5	C24—C23—H23	120.2
H5C—C5—H5D	109.5	C22—C23—H23	120.2
C3—C5—H5E	109.5	C25—C24—C23	119.7 (3)
H5C—C5—H5E	109.5	C25—C24—H24	120.1
H5D—C5—H5E	109.5	C23—C24—H24	120.1
N1—C6—C7	126.7 (3)	C24—C25—C26	118.1 (3)
N1—C6—H6	116.6	C24—C25—H25	120.9
C7—C6—H6	116.6	C26—C25—H25	120.9
C8—C7—C6	121.1 (3)	N3—C26—C25	123.0 (3)
C8—C7—C12	119.7 (3)	N3—C26—H26	118.5
C6—C7—C12	119.2 (3)	C25—C26—H26	118.5
			
O3—Cu1—N1—C6	2.9 (2)	Cu1—O3—C8—C9	168.2 (2)
O1—Cu1—N1—C6	−157.1 (2)	C6—C7—C8—O3	0.9 (5)
N3—Cu1—N1—C6	−107.0 (5)	C12—C7—C8—O3	−176.3 (3)
N2—Cu1—N1—C6	89.3 (2)	C6—C7—C8—C9	177.2 (3)
O3—Cu1—N1—C2	166.36 (19)	C12—C7—C8—C9	−0.1 (4)
O1—Cu1—N1—C2	6.28 (18)	O3—C8—C9—C10	177.9 (3)
N3—Cu1—N1—C2	56.4 (6)	C7—C8—C9—C10	1.4 (5)
N2—Cu1—N1—C2	−107.24 (19)	C8—C9—C10—C11	−1.7 (5)
N1—Cu1—N2—C17	−7.2 (3)	C9—C10—C11—C12	0.6 (5)
O3—Cu1—N2—C17	83.1 (3)	C9—C10—C11—C16	−179.2 (3)
O1—Cu1—N2—C17	−101.3 (3)	C10—C11—C12—C13	−179.5 (3)
N3—Cu1—N2—C17	175.9 (3)	C16—C11—C12—C13	0.3 (4)
N1—Cu1—N2—C21	−169.10 (19)	C10—C11—C12—C7	0.7 (4)
O3—Cu1—N2—C21	−78.8 (2)	C16—C11—C12—C7	−179.5 (3)
O1—Cu1—N2—C21	96.8 (2)	C8—C7—C12—C11	−0.9 (4)
N3—Cu1—N2—C21	14.08 (19)	C6—C7—C12—C11	−178.2 (3)
N1—Cu1—N3—C26	6.4 (7)	C8—C7—C12—C13	179.3 (3)
O3—Cu1—N3—C26	−103.6 (2)	C6—C7—C12—C13	2.0 (4)
O1—Cu1—N3—C26	56.1 (2)	C11—C12—C13—C14	0.6 (4)
N2—Cu1—N3—C26	171.1 (2)	C7—C12—C13—C14	−179.6 (3)
N1—Cu1—N3—C22	−174.3 (4)	C12—C13—C14—C15	−1.2 (5)
O3—Cu1—N3—C22	75.7 (2)	C13—C14—C15—C16	0.8 (5)
O1—Cu1—N3—C22	−124.6 (2)	C14—C15—C16—C11	0.1 (5)
N2—Cu1—N3—C22	−9.6 (2)	C12—C11—C16—C15	−0.6 (5)
N1—Cu1—O1—C1	2.9 (2)	C10—C11—C16—C15	179.1 (3)
O3—Cu1—O1—C1	−75.7 (3)	C21—N2—C17—C18	−1.6 (5)
N3—Cu1—O1—C1	−168.7 (2)	Cu1—N2—C17—C18	−162.2 (3)
N2—Cu1—O1—C1	117.1 (2)	N2—C17—C18—C19	1.8 (6)
N1—Cu1—O3—C8	12.5 (2)	C17—C18—C19—C20	−0.7 (6)
O1—Cu1—O3—C8	89.6 (3)	C18—C19—C20—C21	−0.5 (6)
N3—Cu1—O3—C8	−177.8 (2)	C17—N2—C21—C20	0.3 (5)
N2—Cu1—O3—C8	−102.1 (2)	Cu1—N2—C21—C20	164.7 (3)
Cu1—O1—C1—O2	171.2 (2)	C17—N2—C21—C22	179.0 (3)
Cu1—O1—C1—C2	−11.4 (3)	Cu1—N2—C21—C22	−16.6 (3)
C6—N1—C2—C1	152.0 (3)	C19—C20—C21—N2	0.7 (5)
Cu1—N1—C2—C1	−12.6 (3)	C19—C20—C21—C22	−177.9 (3)
C6—N1—C2—C3	−86.9 (3)	C26—N3—C22—C23	1.5 (4)
Cu1—N1—C2—C3	108.5 (2)	Cu1—N3—C22—C23	−177.8 (2)
O2—C1—C2—N1	−166.8 (3)	C26—N3—C22—C21	−176.8 (3)
O1—C1—C2—N1	15.7 (4)	Cu1—N3—C22—C21	3.8 (3)
O2—C1—C2—C3	69.5 (3)	N2—C21—C22—N3	10.1 (4)
O1—C1—C2—C3	−108.0 (3)	C20—C21—C22—N3	−171.2 (3)
N1—C2—C3—C5	64.6 (4)	N2—C21—C22—C23	−168.1 (3)
C1—C2—C3—C5	−175.1 (3)	C20—C21—C22—C23	10.6 (5)
N1—C2—C3—C4	−63.2 (4)	N3—C22—C23—C24	0.6 (5)
C1—C2—C3—C4	57.2 (4)	C21—C22—C23—C24	178.7 (3)
C2—N1—C6—C7	−179.3 (3)	C22—C23—C24—C25	−2.1 (5)
Cu1—N1—C6—C7	−16.7 (4)	C23—C24—C25—C26	1.6 (5)
N1—C6—C7—C8	16.5 (5)	C22—N3—C26—C25	−2.1 (5)
N1—C6—C7—C12	−166.3 (3)	Cu1—N3—C26—C25	177.3 (2)
Cu1—O3—C8—C7	−15.5 (4)	C24—C25—C26—N3	0.5 (5)

### Hydrogen-bond geometry (Å, º)

**Table:**

*D*—H···*A*	*D*—H	H···*A*	*D*···*A*	*D*—H···*A*
O4—H4*A*···O2^i^	0.85	1.99	2.823 (3)	168
O4—H4*B*···O3	0.85	2.00	2.847 (3)	172
O5—H5*A*···O2^ii^	0.85	2.07	2.870 (3)	158
O5—H5*B*···O6^iii^	0.85	2.04	2.852 (4)	160
O6—H6*A*···O4^iv^	0.85	2.10	2.900 (4)	156
O6—H6*B*···O5^v^	0.85	1.97	2.814 (4)	174

Symmetry codes: (i) *x*−1, *y*, *z*; (ii) *x*, *y*, *z*+1; (iii) *x*, *y*−1, *z*; (iv) −*x*+1, −*y*+1, −*z*+1; (v) −*x*+2, −*y*+1, −*z*+2.
